# A closer look into the microbiome of microalgal cultures

**DOI:** 10.3389/fmicb.2023.1108018

**Published:** 2023-01-26

**Authors:** Pia Steinrücken, Steve Jackson, Oliver Müller, Pål Puntervoll, Dorinde M. M. Kleinegris

**Affiliations:** ^1^Department of Biological Sciences, University of Bergen, Bergen, Norway; ^2^NORCE Climate & Environment - NORCE Norwegian Research Centre AS, Bergen, Norway

**Keywords:** microalgal cultures, microbiome, bacterial community composition, Illumina sequencing, growth medium

## Abstract

Although bacteria are commonly co-occurring in microalgal cultivation and production systems, little is known about their community structure and how it might be affected by specific microalgal groups or growth conditions. A better understanding about the underlying factors that determine the growth of specific bacterial populations is not only important for optimizing microalgal production processes, but also in the context of product quality when the algal biomass is to be used for future food or feed. We analyzed the bacterial community composition associated with nine microalgal strains in stock culture, maintained in two different growth media, to explore how specific taxonomic microalgal groups, microalgal origin, or the growth medium affect the bacterial community composition. Furthermore, we monitored the bacterial community composition for three *Phaeodactylum* strains during batch cultivation in bubble columns to examine if the bacterial composition alters during cultivation. Our results reveal that different microalgal genera, kept at the same cultivation conditions over many years, displayed separate and unique bacterial communities, and that different strains of the same genus had very similar bacterial community compositions, despite originating from different habitats. However, when maintained in a different growth medium, the bacterial composition changed for some. During batch cultivation, the bacterial community structure remained relatively stable for each *Phaeodactylum* strain. This indicates that microalgae seem to impact the development of the associated bacterial communities and that different microalgal genera could create distinct conditions that select for dominance of specific bacteria. However, other factors such as the composition of growth medium also affect the formation of the bacterial community structure.

## 1. Introduction

Microalgae are considered an important new and sustainable feedstock for different food and non-food commodities. They are rich in valuable compounds such as omega-3 fatty acids, proteins, carbohydrates, carotenoids, vitamins, and minerals and their production offers many sustainability features. In the last decades, microalgae have established a multi-million dollar industry and are being produced for food, feed, and health-and cosmetic-related products (Fernández et al., 2021). In cultivation systems, microalgae co-exist together with bacteria, which either have been associated with the microalgae since isolation or they have been introduced later through air or non-sterile handling. Only in a very few cases, and at a small scale, can microalgae in culture be maintained axenically (Biondi et al., 2018; Lian et al., 2021).

In natural aquatic environments, interactions between bacteria and microalgae represent a fundamental ecological relationship, influencing carbon and nutrient cycling and regulating the productivity and stability of aquatic food webs (Seymour et al., 2017). The heterotrophic bacteria obtain a large fraction of their carbon demand from microalgae. Microalgae release dissolved organic carbon (DOC) into their immediate surrounding, the phycosphere, which is colonized by bacteria (Lian et al., 2021). Many bacteria, in turn, promote microalgal growth by providing limiting nutrients *via* remineralization, or synthesis of special components such as vitamins, thus enhancing micronutrient availability (Seymour et al., 2017). Other bacteria can have a negative impact on microalgae by competing for inorganic nutrients or may even kill or lyse microalgal cells (Paul and Pohnert, 2011; Wang et al., 2016; Aiyar et al., 2017). Bacteria also process microalgae-derived particulate organic matter or dead cells and contribute in releasing DOC into the surrounding environment (Schäfer et al., 2002).

Associated bacteria in microalgal production systems have only recently gained particular interest, as several studies have revealed that microalgal production can be affected by the associated bacteria, and that some bacterial strains can significantly increase, while others can inhibit microalgal growth or metabolite production (Le Chevanton et al., 2013; Biondi et al., 2018; Berthold et al., 2019; Ling et al., 2020; Chorazyczewski et al., 2021; González-González and De-Bashan, 2021; Lian et al., 2021). Different microalgae might create special niches for specific bacteria and the successive transfer of microalgal cultures could select for certain bacterial populations to grow in association with a particular microalgae (Schäfer et al., 2002). However, a deeper understanding on whether bacterial communities are associated with different microalgal groups, growth conditions or growth systems, and what causes the development of specific bacterial populations is still lacking.

In this study, we aimed to elucidate if the bacterial community composition is related to specific factors such as microalgal taxonomy, origin of isolation, growth medium, or growth phases. Therefore, in a first analysis, the microbiome of 18 microalgal stock cultures, covering nine strains from three different genera (*Phaeodactylum*, *Entomoneis*, and *Tetraselmis*) was studied. The strains originated from various locations and habitats, were isolated at different times, and were each maintained in two different growth media. In a subsequent bubble column experiment, the microbiome was monitored during batch cultivation for three different *Phaeodactylum* strains to investigate if the bacterial communities alter during cultivation and with different growth phases.

## 2. Methods

### 2.1. Cultivation conditions of stock cultures

All microalgal strains investigated in this study were kept as stock cultures in 15 ml glass tubes at 7°C and an irradiance of 30 μmol m^−2^ s^−1^ with a light:dark cycle of 16:8. Each microalgal strain has been maintained in both, modified Conway medium (Walne, 1970) and NORCE medium. The NORCE medium was designed in our labs for pilot-scale microalgal production and contains much higher amounts of nitrate and phosphate (12.5 and 0.88 mmol L^−1^ respectively, with KH_2_PO_4_ as phosphate source) compared to Conway medium (1.2 and 0.11 mmol L^−1^, respectively, with NaH_2_PO_4_ as phosphate source), allowing the microalgae to grow to higher densities without reaching nutrient limitation. In addition, NORCE medium uses a commercial trace metal mix (YaraTera REXOLIN APN), which makes it easier and cheaper to prepare. Both media were prepared with natural sterilized seawater (35 ppt) and addition of 0.2 μm filtered stock solutions. The final concentration of the nutrient composition for both media is shown in the Supplementary section (Supplementary Table S1). Cultures were transferred to fresh medium every 3 months, by inoculating an aliquot of the culture suspension into fresh Conway or NORCE medium, respectively, using sterilized pipettes.

### 2.2. The microbiome of different microalgal stock cultures

**Figure 1:**
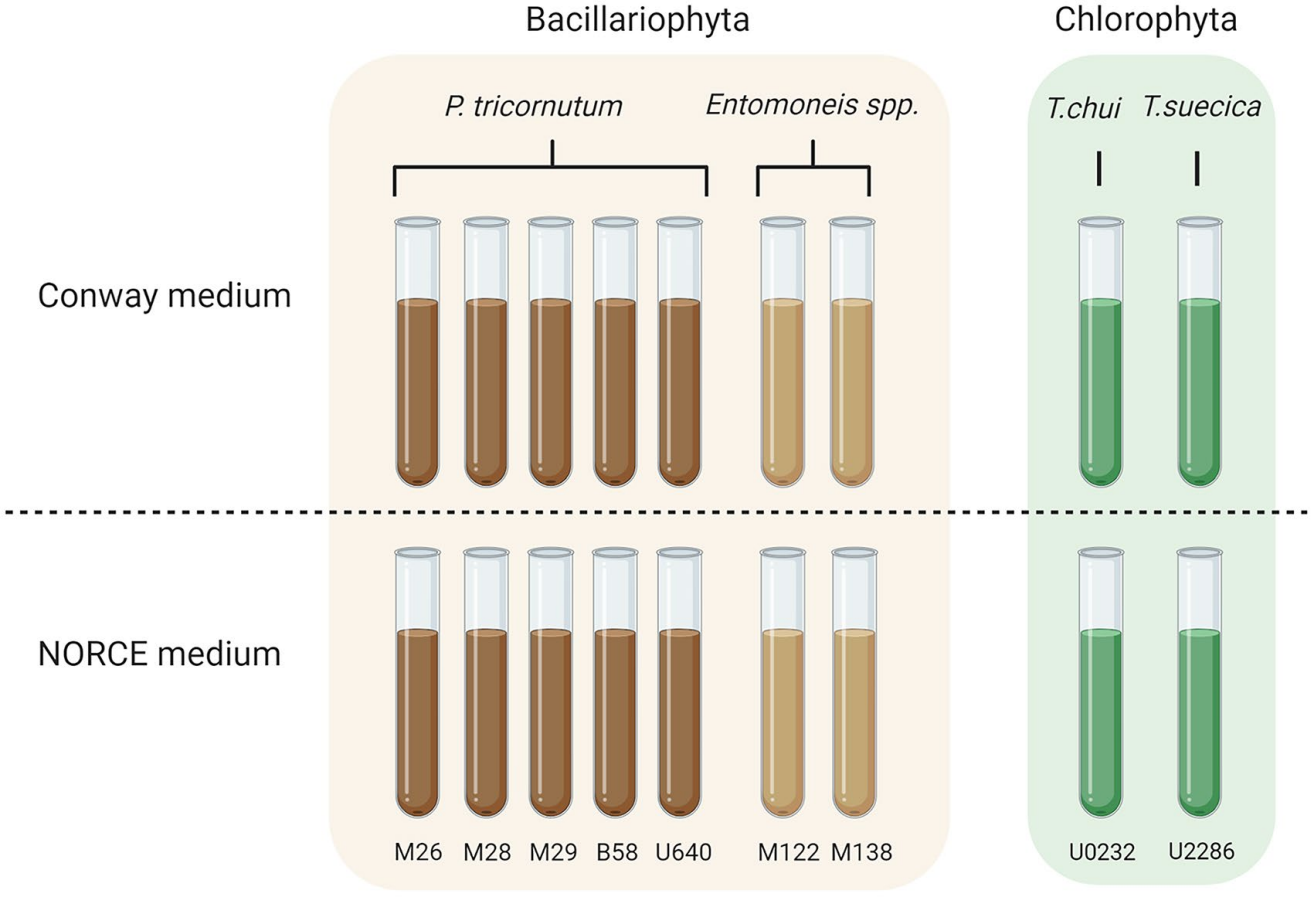
Overview over stock cultures that were screened for bacterial community composition. All strains were kept in two different media (Conway and NORCE) for different time periods before sampling. Figure created with BioRender.com.

Nine separate microalgal strains were selected from our stock culture collection for analysis of the bacterial community composition, including five strains of *Phaeodactylum tricornutum*, two strains of *Entomoneis* sp., one *Tetraselmis chui* strain, and one *Tetraselmis suecica* strain. These nine strains cover four different microalgal species, three genera, and two phyla, and have been maintained in two different growth media (Conway and NORCE) for several months or years (Figure 1). The selected strains are fast growing with high nutritional value, and are either already used in industrial production (*Tetraselmis and Phaeodactylum*) or being considered for possible future production or as feed in aquaculture hatcheries (*Entomoneis*; Knuckey et al., 2002; Steinrücken et al., 2017). Four of the *Phaeodactylum* strains (M26, M28, M29, and B58) and the two *Entomoneis* strains (M122 and M138) were isolated from local coastal environments in the Bergen area (Norway) and have at the time point of microbiome analysis been maintained in our laboratory in Conway medium for 23 years (B58; Prestegard et al., 2009) or 6 years (the others; Steinrücken et al., 2017) and in NORCE medium for 16 months. *Phaeodactylum* strain UTEX 640 and the two *Tetraselmis* species (*T. chui* UTEX LB 232, *T. suecica* UTEX LB 2286) were obtained from the Culture Collection of Algae at the University of Texas at Austin (UTEX) and have been maintained in our laboratories in Conway medium for 4 years and in NORCE medium for 16 months (UTEX 640) or 2 months (*Tetraselmis* species). For simplicity, strains from culture collection were assigned a short name. Strain *Phaeodactylum* U640 was originally obtained as axenic culture (Table 1).

**Table 1:** Overview over stock cultures that were screened for bacterial community composition.

Species	Strain	Short name	Phylum	Isolation site	Maintained in	
					Conway	NORCE
*P. tricornutum*	M26*	M26	Bacillariophyta	Store Lungegårdsvann Bergen, Norway	6 years	16 months
*P. tricornutum*	M28*	M28	Bacillariophyta	Puddefjorden Bergen Norway	6 years	16 months
*P. tricornutum*	M29*	M29	Bacillariophyta	Puddefjorden Bergen Norway	6 years	16 months
*P. tricornutum*	B58*	B58	Bacillariophyta	Puddefjorden Bergen Norway	23 years	16 months
*P. tricornutum*	UTEX 640^A^	U640	Bacillariophyta	England, UK^X^	4 years	16 months
*Entomoneis* sp.	M122*	M122	Bacillariophyta	Store Lungegårdsvann Bergen, Norway	6 years	16 months
*Entomoneis* sp.	M138*	M138	Bacillariophyta	Store Lungegårdsvann Bergen, Norway	6 years	16 months
*T. chui*	UTEX LB 232	Tchui	Chlorophyta	Scotland	4 years	2 months
*T. suecica*	UTEX LB 2286	Tsue	Chlorophyta	La Spezia, Italy	4 years	2 months

Strains with an asterisk (*) were isolated from local coastal environments in the Bergen area, others were obtained from culture collection (UTEX). Strain with an (A) was classified axenic in the culture collection. Isolation site with an (X) is not mentioned in the respective culture collection but for the deposition CCAP 1052/1B at the Culture Collection of algae and protozoa. All strains were kept in two different media (Conway and NORCE) for different time periods before sampling.

Three weeks before sampling for bacterial community composition, the 18 microalgal stock cultures (9 strains in two different growth media) were newly transferred to 15 ml glass tubes with fresh medium. Cultures were kept at the same growth conditions as described in 2.1 and were additionally vortexed every workday to maintain homogenous cultures and prevent the algae from attaching to the glass tubes. After 3 weeks, samples were taken from each culture for (i) microbiome analyses (one single sample each) and (ii) algal and bacterial cell concentration measurements by flow cytometry (three technical replicates each). For microbiome composition, one single analysis was performed for each sampling, as previous analyses have shown high similarity between technical replicates (Wen et al., 2017; Marotz et al., 2019). However, one sample was analyzed in duplicate (*Phaeodactylum* strain B58, NORCE medium) for quality control of the sequencing and sample preparation.

### 2.3. The microbiome of three *Phaeodactylum* strains during batch cultivation in bubble columns

**Figure 2:**
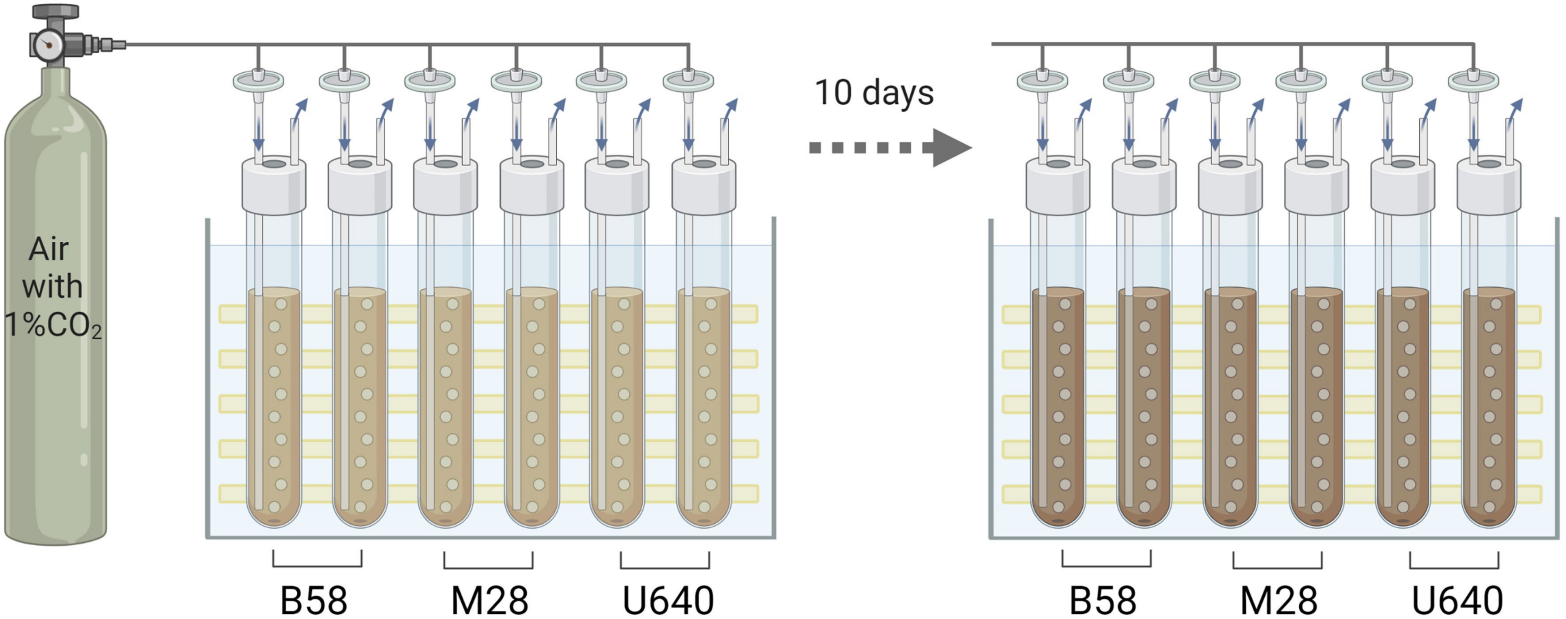
Experimental setup for batch experiment in bubble columns. Batch cultures of three *Phaeodactylum tricornutum* strains were grown at controlled growth conditions until stationary phase was reached, with 2 biological replicates for each strain. Cultures were kept at a temperature of 24°C, bubbled with air enriched with 1% CO_2_, and irradiance was increased from 30 to 500 μmol m^−2^ s^−1^ with increasing biomass concentration. Samples for optical density were taken daily and for cell count and microbiome analysis on Days 0, 5, and 9 (U640) or 10 (B58, M28). Figure created with BioRender.com.

Three *Phaeodactylum* strains (B58, M28, and U640) were first upscaled by transferring biomass from the respective NORCE stock culture to 200 ml sterile NORCE medium and incubated at 15°C and 50 μmol m^−2^ s^−1^ (light:dark cycle of 16:8) for 2 weeks. Thereafter, biomass of each strain was centrifuged (2,264 g, 5 min) and resolved in 250 ml fresh NORCE medium to an optical density (OD, 750 nm) of approximately 0.1. Each 250 ml inoculum was distributed into two 80 ml glass tubes (two biological replicates for each strain), which were sealed with a rubber top. The glass tubes were placed in a Multi-Cultivator MC 1000-OD with a temperature-controlled water bath and LED lights in the back (Figure 2). For carbon supply and culture mixing, 0.2 μm filtered air, enriched with 1% CO_2_, was bubbled through glass capillaries into the bottom of each glass tube and the temperature was kept constant at 24°C. Irradiance (24 h) was increased accordingly with the increase in biomass concentration (based on daily measurements of OD 750) from 30 to a maximum of 500 μmol m^−2^ s^−1^ (Supplementary section, Supplementary Table S2) to avoid photoinhibition at the beginning of the cultivation and light limitation during linear growth. Cultures were grown until stationary phase was reached (day 9 for U640 and day 10 for B58 and M28). Samples were taken with a syringe from a port connected to a glass capillary leading into the culture to facilitate sterile sampling. Samples for OD were taken daily (two technical replicates per biological duplicate) to follow growth of the cultures. Samples for bacterial community analysis (one single sample per biological duplicate) and algal and bacterial cell concentration (three technical replicates per biological duplicate) were taken on Day 0 from the 250 ml inoculum and on days 5 (linear growth phase) and 9 or 10 (stationary phase) from each tube.

### 2.4. Analytical procedures

#### 2.4.1. Optical densities

Optical densities were measured at 750 nm with diluted samples to give an attenuation below 0.2 with a spectrophotometer (UV-2401 PC, Shimadzu Corporation, Kyoto, Japan).

#### 2.4.2. Bacterial and microalgal abundance using flow cytometry

To determine bacterial and microalgal cell concentrations, culture volumes of 0.5 ml were transferred into 2 ml cryotubes, fixed with glutaraldehyde (0.5% final conc.) at 4°C for a minimum of 30 min, flash frozen in liquid nitrogen, and stored at −80°C until analysis. Samples were measured on an Attune Acoustic Focusing Flow Cytometer (Applied Biosystems by Life technologies). For bacterial counts, frozen samples were thawed and diluted × 1,000–10,000 (dependent on culture density) with 0.2 μm filtered TE buffer (Tris 10 mM, EDTA 1 mM, pH 8) and stained with a green fluorescent nucleic acid dye (SYBR Green I; Molecular Probes, Eugene, Oregon, United States). After 10 min of dark incubation, 100 μl of each sample was counted at medium flow rate (100 μl min^−1^). For microalgal counts, thawed samples were diluted × 10–1,000 (dependent on culture density) with 0.2 μm filtered seawater, and a volume of 500 μl was counted at high flow rate (500 μl min^−1^).

#### 2.4.3. Microbiome – DNA extraction, amplification, and preparation for Illumina sequencing

A total of 37 samples were analyzed for bacterial community composition, covering 19 samples from stock cultures, 15 samples from the batch culture experiment, and three controls (filter blank, Conway medium, NORCE medium). Culture samples were filtered onto a 0.22 μm Durapore® Membrane Filter (Ø 47 mm, Merck-Millipore). The filters were folded with sterilized tweezers and transferred into 2 ml cryotubes and stored at −80°C until analysis. For stock cultures, a volume of 3 ml was filtered, while for the batch experiment, 20 ml was filtered on Day 0, and 3 ml for linear and stationary phase samples. Furthermore, a blank filter and two control filters with 50 ml of Conway and 50 ml of NORCE medium, respectively, were sampled. DNA from the filters were extracted using DNeasy Power Soil Kit (Qiagen), following manufacturer’s instructions with minor modifications. Instead of 675 μl, 650 μl was loaded onto a MB Spin Column after Solution C4 was added, and instead of 100 μl, 80 μl of Solution C6 (heated to 70°C) was added to the filter membrane followed by 10 min incubation. Extracted DNA was stored at −20°C until PCR amplification. Amplification of the bacterial 16S rRNA gene V5-V7 region was performed using a two-step nested PCR approach. The first PCR step was performed in triplicates using the chloroplast-excluding primers 799F (5′-AACMGGATTAGATACCCKG-3′) and 1193R (5′-ACGTCATCCCCACCTTCC-3′; Beckers et al., 2016). To reach a reaction volume of 20 μl, 10 ng DNA, 10 μl HotStarTaq Master Mix (Qiagen), and 0.5 μM of each primer were mixed with nuclease-free water. PCR reaction conditions were as follows: initial denaturation of 15 min at 95°C, followed by 25 cycles of 95°C for 20 s, 53°C for 30 s, and 72°C for 45 s and a final extension step of 72°C for 7 min. Triplicate PCR products were pooled and quantified using Qubit 3.0 Fluorometer. For the second PCR, each sample was designated to a specific forward and reverse primer combination, each containing a unique eight-nucleotide barcode. Volumes of 10 ng pooled PCR product were combined with 25 ml HotStarTaq Master Mix, 0.5 μM of each respective nested primer, and nuclease-free water to a reaction volume of 50 μl. PCR reaction conditions were as follows: initial denaturation of 15 min at 95°C, followed by 12 cycles of 95°C for 20 s, 62°C for 30 s, and 72°C for 30 s, followed by and a final extension step of 72°C for 7 min. The final PCR products were purified with Agencourt AMPure XP Beads (Beckman Coulter Inc., CA, United States) and pooled in equimolar concentrations. The amplicon pool was quantified using Qubit 3.0 Fluorometer and the quality was assessed by agarose gel electrophoresis. The PCR products were sequenced at the Norwegian Sequencing Center (Oslo, Norway) using the MiSeq platform (MiSeq Reagent Kit v3, Illumina, CA, United States). Illumina sequencing data are available at the European nucleotide archive (ENA) under study accession number PRJEB46865.

#### 2.4.4. Microbiome – Bioinformatic sequence analysis

The retrieved paired-end sequence data from Illumina Miseq v3 sequencing (12.9 M reads, *ca.* 103,000 reads per sample) were processed using the R package DADA2 version 1.18.0 (Callahan et al., 2016, Divisive Amplicon Denoising Algorithm 2, RRID:SCR_008205) in R version 4.0.3 (R Core Team, 2020). Sequence reads were trimmed and filtered based on quality scores, before sequences were dereplicated and Amplicon sequencing variants (ASVs, referred to as operational taxonomic units [OTUs] for simplicity) inferred. Both forward and reverse reads were denoised and merged before chimeric sequences were removed. Taxonomy was assigned using the 16S silva database version132 (RRID:SCR_006423). Bacterial 16S rRNA genes were successfully sequenced for their V5-V7 hypervariable region and using the primer pair 799f-1193r successfully excluded chloroplast DNA during amplification, and low amounts of mitochondria and eukaryotic DNA were amplified. Obtained sequencing reads were similar for both experiments with an average of 99,752 (input), 27,644 (after quality filtering, merging and exclusion of chimeric sequences), and 27,451 (excluding mitochondria and eukaryotes) reads, showing that high-quality sequencing was accomplished. All three control samples (filter blank, Conway medium, NORCE medium) were with only 709–1,594 reads (183–390 after excluding chimeric sequences) negative, indicating no contamination throughout the workflow. Bacteria were identified to the genus level and in general, reads of different OTUs representing the same bacterial genus were merged, except for *Marinobacter*, where one OTU was specific for Diatoms and one for *Tetraselmis* sp. and was therefore separated into *Marinobacter*_1 and *Marinobacter*_2, respectively.

### 2.5. Statistics

To test if the observed differences in bacterial and algal cell concentrations between the two growth media and three microalgal genera (stock culture screening) and between the sampling time points and biological replicates (bubble column experiment) were significant, two-way ANOVA was performed. The bacterial community composition datasets of both experiments were analyzed by hierarchical clustering using calculated Aitchison distances and principal component analysis (PCA), performed on data normalized using centered log-ratio transformation (Gloor and Reid, 2016). Furthermore, a statistical analysis of the dissimilarities between and within microalgal genus groups (stock culture screening) and between and within the three *Phaeodactylum* strains (bubble column experiment) was done with ANOSIM using the Aitchison distances. For both experiments, microalgal and bacterial cell counts were also used to perform hierarchical clustering of the samples, using binomial deviance dissimilarity measure distance calculation. Finally, a Mantel test was performed to test for correlation between the two dissimilarity matrices (microbiome and cell counts). Two-way ANOVA was done using GraphPad Prism 9 (RRID:SCR_002798), and all other statistical analyses were done using the R package vegan (RRID:SCR_011950). Coordinates of the scores and loadings of the PCA plot were illustrated using GraphPad Prism 9.

## 3. Results

### 3.1. Microbiome analysis of microalgal stock cultures

#### 3.1.1. Algal and bacterial cell concentrations

**Figure 3:**
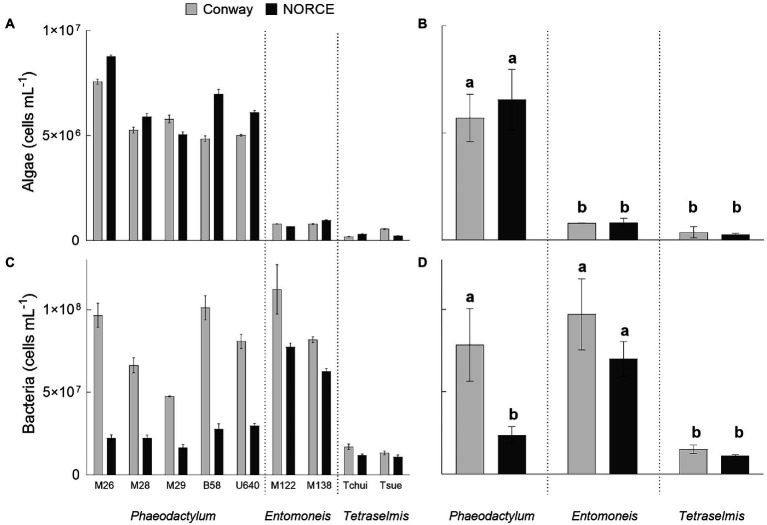
Algal and bacterial cell counts for stock cultures of nine microalgal strains in two different media (Conway and NORCE). **(A)** Algal cell concentration for each culture. **(B)** Average algal cell concentration for cultures grouped by microalgal genera and medium. **(C)** Bacterial cell concentration for each culture. **(D)** Average bacterial cell concentration for cultures grouped as in **B**. **(A,C)** Show average and standard deviation of three measurement replicates. **(B,D)** Show average and standard deviation of the different cultures. Same letters above the bars indicate no significant differences (*p* < 0.05).

As a first step of the analysis, we determined the algal and bacterial cell concentrations of each stock culture (Figures 3A,C). The microalgal cell concentrations ranged from 1.9 × 10^5^ to 8.8 × 10^6^ cells mL^−1^, and in every stock culture, the bacterial concentrations were higher, ranging from 1.1 × 10^7^ to 1.1 × 10^8^ cells mL^−1^. Bacteria were also detected in *Phaeodactylum* strain U640, which was originally obtained as an axenic strain from culture collection. The bacteria-to-algae ratio was generally higher in Conway compared to NORCE medium, and *Phaeodactylum* strains had the lowest bacteria-to-algae ratios with values between 3 and 21, *Tetraselmis* strains between 24 and 92, and *Entomoneis* strains between 65 and 141.

The microalgal cell concentrations of stock cultures from the same genera were at comparable levels but differed between genera (Figure 3A). In both media, microalgal cell concentrations were significantly higher for *Phaeodactylum* strains (average of 5.7 and 6.6 × 10^6^ cells mL^−1^ for Conway and NORCE medium, respectively), compared to *Entomoneis* (average of 8 and 8.1 × 10^5^ cells mL^−1^) and *Tetraselmis* strains (average of 3.7 and 2.7 × 10^5^ cells mL^−1^; Figure 3B). When comparing the microalgal cell concentrations of Conway and NORCE medium stock cultures of the same strains, some differences were observed (Figure 3A), but when averaged by genera, these differences were not significant (Figure 3B).

The bacterial cell concentrations of the NORCE medium stock cultures were also comparable within genera but different between genera (Figure 3C). This trend was not as apparent for the Conway medium stock cultures. For the *Phaeodactylum* strain cultures, the bacterial cell numbers differed significantly between the two growth media (average of 7.9 and 2.4 × 10^7^ cells mL^−1^ for Conway and NORCE medium, respectively; Figure 3D). In contrast, no significant differences were found neither for the *Entomoneis* (average of 9.7 and 7 × 10^7^ cells mL^−1^) nor the *Tetraselmis* cultures (average of 1.5 and 1.1 × 10^7^ cells mL^−1^).

#### 3.1.2. Bacterial community composition

**Figure 4:**
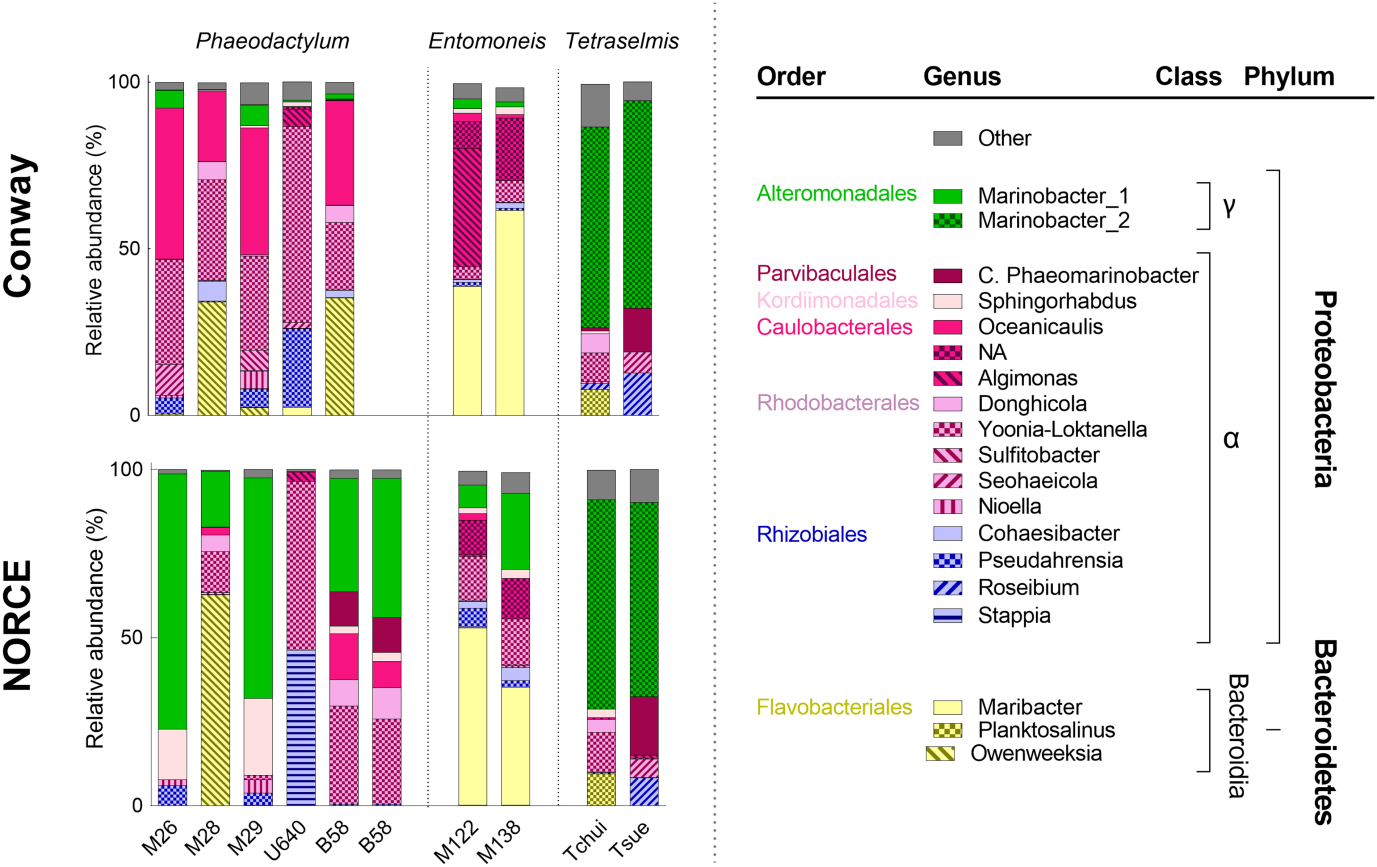
Bacterial community composition for stock cultures of nine microalgal strains in Conway and NORCE medium. Genera with an abundance higher than 5% of total bacterial abundance are shown, bacteria with lower abundance are summarized in “Other.”

Microbiome analysis of the stock cultures revealed a total of 116 bacterial OTUs, representing 64 different bacterial genera, and the bacterial community composition for the nine strains in Conway and NORCE media is shown in Figure 4. The most dominant bacterial genera present in the microalgal cultures (>97%) belonged to the classes *Alphaproteobacteria*, *Gammaproteobacteria* (phylum Proteobacteria), and *Bacteroidia* (phylum Bacteroidetes). The duplicate analysis of B58 (NORCE medium), which served as a quality control of the microbiome analysis procedure, produced very similar bacterial communities, suggesting consistent sample preparation and sequencing results (Figure 4). In Conway medium, the bacterial diversity in microalgal stock cultures varied strongly between the different microalgal genera but was similar for the different strains within each genus. In NORCE medium, the bacterial diversity was similar for the two *Tetraselmis* species and for the two *Entomoneis* strains but varied greatly between the different *Phaeodactylum* strains. The only exception were *Phaeodactylum* strains M26 and M29, which had very similar bacterial community compositions in the NORCE medium. In both media, the bacterial composition in the U640 stock cultures differed the most from the remaining *Phaeodactylum* strains. For both *Tetraselmis* species, the bacterial structure in NORCE medium was nearly identical to the one in Conway medium. Also, both *Entomoneis* strains had very similar bacterial compositions in the two media but differed in their relative abundances. The five *Phaeodactylum* strains had greater differences in their bacterial communities in NORCE compared to Conway medium, particularly due to a strong increase in the relative abundance of *Marinobacter_1* in four of the five strains (not U640) in NORCE medium, which was only present in low amounts in Conway medium, and a decrease of different *Alphaproteobacteria* genera which were dominant in Conway medium.

#### 3.1.3. Statistical analysis of bacterial community structure and cell counts

**Figure 5:**
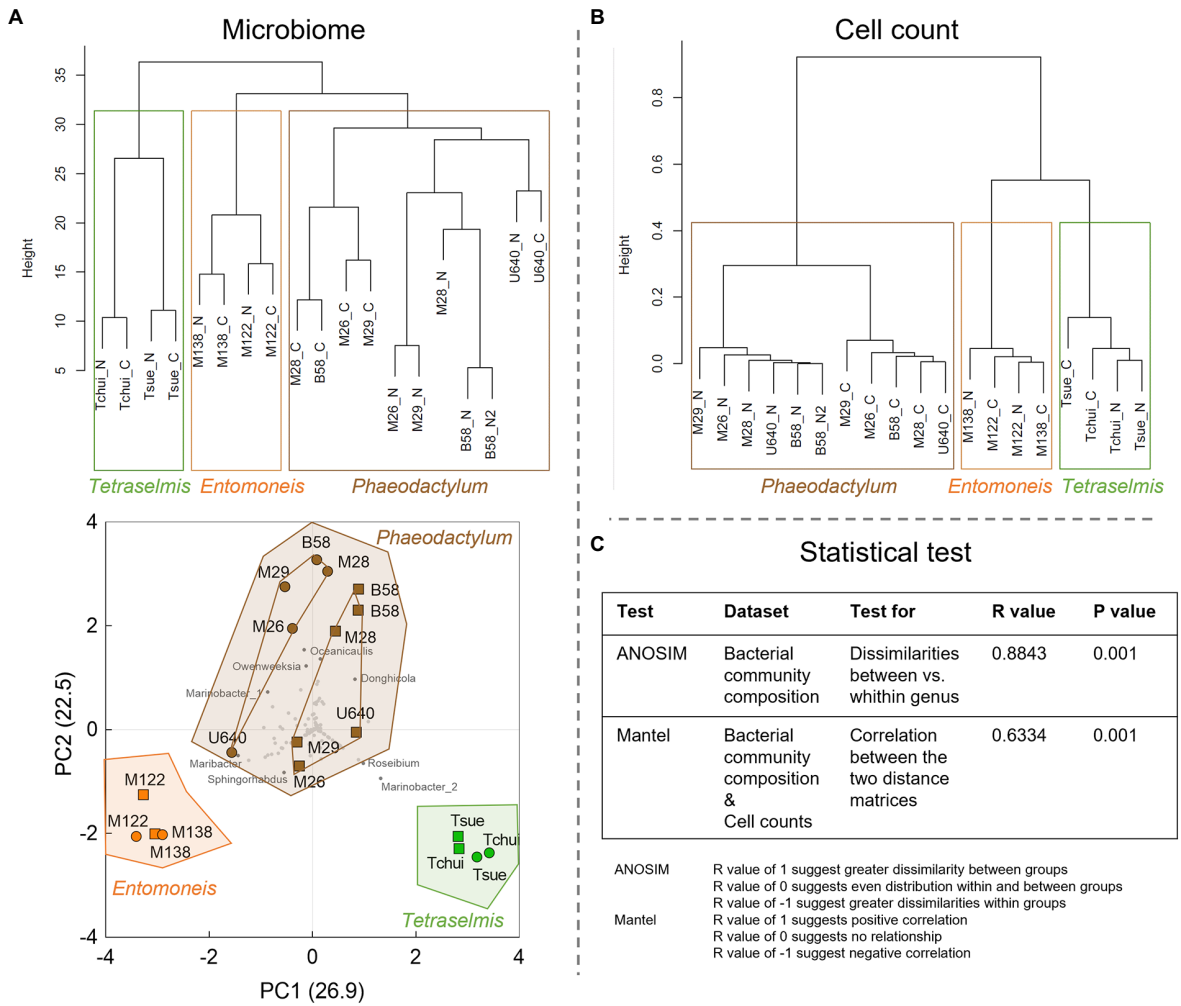
Statistical analysis of the microbiome and cell count data from the stock culture screening. **(A)** Dendrogram (upper) and principal component analysis (PCA, lower) of the normalized bacterial community composition data. All sequenced OTUs were included in the calculations and are shown as gray dots in the PCA plot, and OTUs with largest impact are shown as dark gray dots with bacterial genus name. In the dendrogram, N stands for NORCE and C for Conway medium. In the PCA plot, circles indicate Conway and squares NORCE medium. For *Phaeodactylum* stock cultures inner connecting lines indicate two distinct subclusters defined by culture medium (Conway or NORCE). **(B)** Hierarchical dendrogram of the cell count data (microalgae and bacteria). **(C)** Statistical tests with R and *p* values.

To further explore similarities and differences of the bacterial community structures, the bacterial OTU abundances for all culture stocks were analyzed by hierarchical clustering (Figure 5A). This revealed that the bacterial community structures were more similar within each microalgal genus group than between genera, regardless of culture medium. This was also supported by an analysis of similarities (ANOSIM) test (Figure 5C). For both *Tetraselmis* and *Entomoneis*, the culture stocks clustered by strain, whereas for *Phaeodactylum*, the culture stocks clustered primarily by culture medium (except for strain U640). A PCA of the bacterial community compositions also revealed that the stock cultures clustered by microalgal genera (Figure 5A). In line with the hierarchical clustering results, the *Phaeodactylum* stock cultures also formed two distinct subclusters defined by culture medium (Conway or NORCE). Hierarchical clustering of the culture stocks based on microalgal and bacterial cell counts also revealed distinct clusters for each of the three microalgal genera, and a distinct grouping by growth medium within the *Phaeodactylum* genus (Figure 5B). The two distance matrices used for hierarchical clustering of bacterial community composition and cell counts, respectively, displayed significant correlation (Mantel test, Figure 5C), suggesting that the two measures corroborate each other.

### 3.2. Microbiome analysis of three *Phaeodactylum* strains during batch cultivation in bubble columns

#### 3.2.1. Algal and bacterial cell concentrations

**Figure 6:**
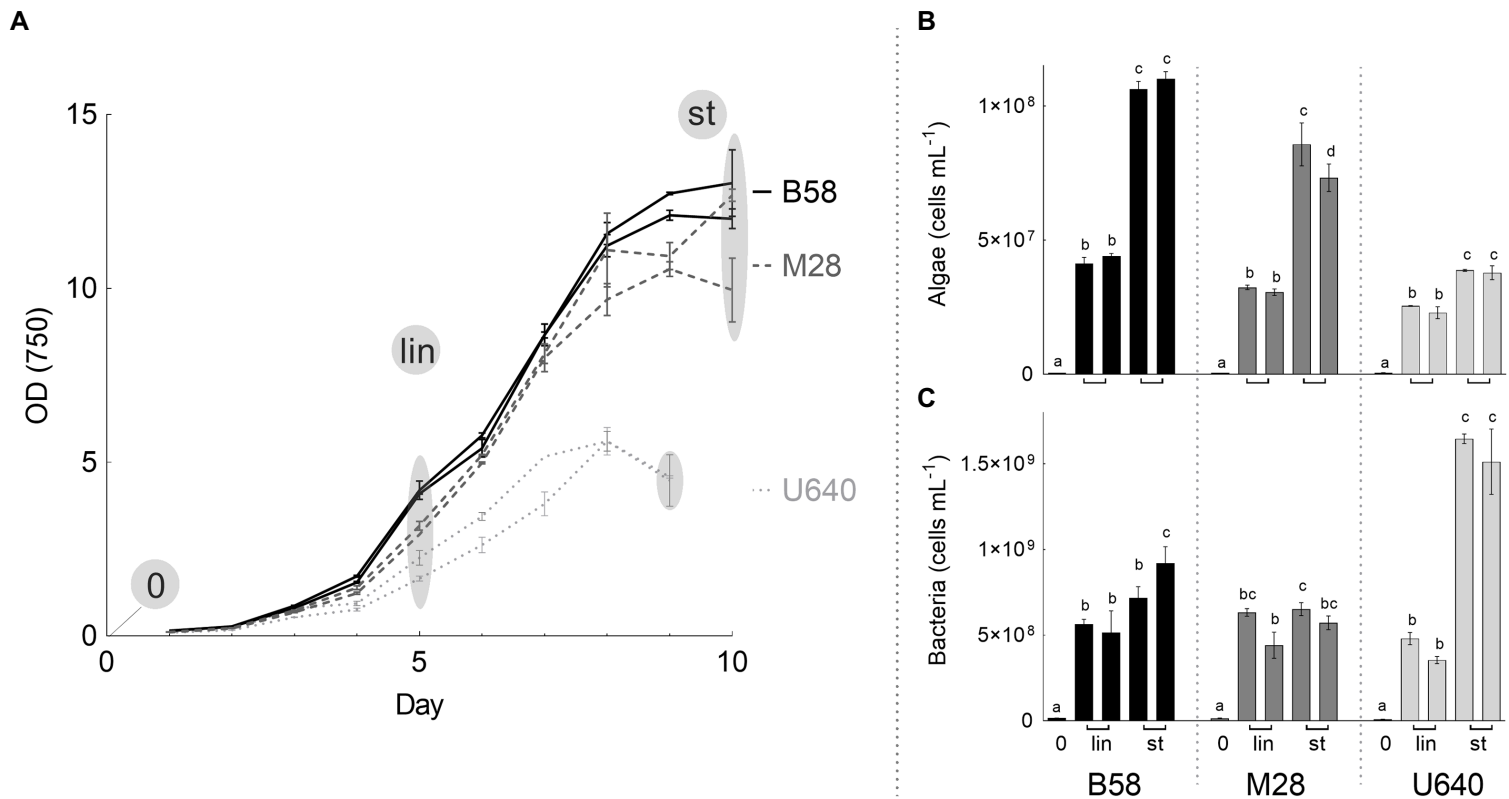
Batch cultivation of three *Phaeodactylum* strains in bubble columns with two biological replicates each. **(A)** OD-based growth curves with the three sampling time points (gray circles) for cell concentration and microbiome analysis, 0 = start of the cultivation, lin: linear growth phase, st: stationary phase. **(B)** Microalgal and **(C)** Bacterial cell concentration at the respective sampling time points. On day 0 samples were taken from the starting culture before being divided in two cultures resulting in one sample for this sampling time point. Values show average and standard deviation of two (OD) or three (cell counts) measurement replicates. For each strain, means of algal or bacterial cell concentrations with the same letter are not significantly different (*p* > 0.05).

Three *Phaeodactylum* strains (B58, M28, and U640) were grown as batch cultures in NORCE medium in bubble columns to investigate how the bacterial community develops during cultivation. Microalgal growth curves (based on OD 750) and cell concentrations for microalgae and bacteria (cells mL^−1^) are shown in Figure 6. Strains B58 and M28 grew faster and reached higher OD values in stationary phase than strain U640, with similar growth patterns for the respective biological replicates (Figure 6A). This was also reflected by the microalgal cell concentrations, which increased to a stronger degree for strains B58 and M28 with 7.3 × 10^7^–1.1 × 10^8^ cells mL^−1^ in stationary phase, compared to strain U640 with 3.8–3.9 × 10^7^ cells mL^−1^ (Figure 6B). The bacterial concentration increased as well with cultivation time for all three strains but to different degrees (Figure 6C). For strains B58 and M28, bacterial cell concentrations had their greatest increase from Day 0 to linear phase, reaching between 4.4 and 6.3 × 10^8^ cells mL^−1^ and a lower, yet not significant increase further to stationary phase yielding 5.7–9.2 × 10^8^ cells mL^−1^. Strain U640 reached bacterial concentrations in linear phase that were comparable to those of B58 and M28 (average 4.2 × 10^8^ cells mL^−1^), but reached a much higher bacterial abundance in stationary phase, with 1.6 × 10^9^ cells mL^−1^. The bacteria-to-algae ratio for strains M28 and B58 decreased from 28 and 24, respectively, at the start of the experiment, to *8 and 7*, respectively, in stationary phase, while for strain U640, the ratio increased from 15 to 41.

#### 3.2.2. Bacterial community composition

**Figure 7:**
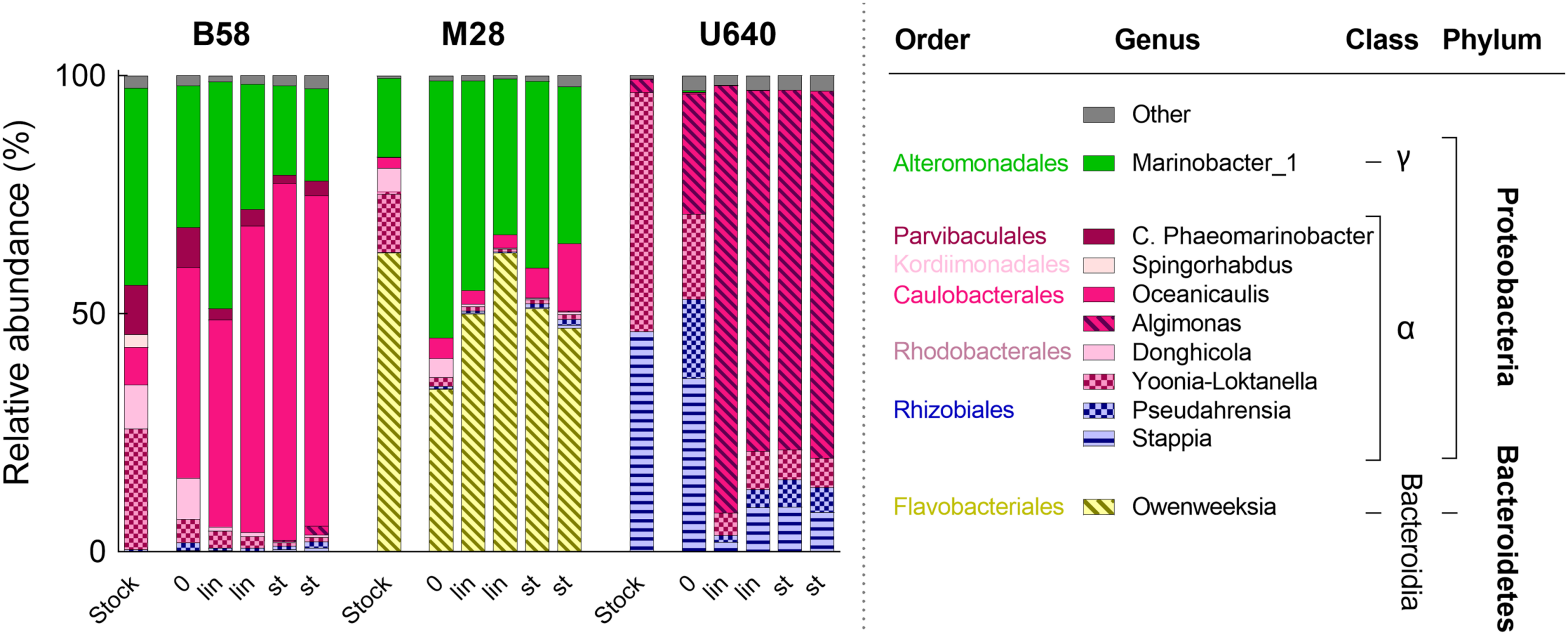
Bacterial community composition during batch cultivation in bubble columns of three *Phaeodactylum* strains in NORCE medium with two biological replicates each. Bacterial genera with an abundance of higher than 5% are shown, bacteria with lower abundance are summarized in “Other.” 0: start of the cultivation, lin: linear growth phase, st: stationary phase. For each strain, the bacterial community composition of the respective NORCE stock culture is shown from Figure 4.

The bacterial community composition for the three strains during batch cultivation (start, linear phase, and stationary phase) is shown in Figure 7. The microbiome analysis revealed a total of 60 bacterial OTUs representing 39 different bacterial genera. For each strain, the bacterial composition during batch cultivation was very similar to that of the parent NORCE stock culture, but the relative abundances of the bacteria changed. For strain B58, *Oceanicaulis* increased in relative abundance from stock culture to the start of the experiment and dominated at the end of the cultivation, while Marinobacter_1 and other genera decreased in relative abundance. For strain M28, *Marinobacter*_1 increased in relative abundance and together with *Owenweeksia* dominated during batch cultivation. Strain U640 mainly contained *Stappia* and *Yoonia-Loktanella* in stock culture. At the start of the batch experiment, *Pseudahrensia* and *Algimonas* had increased in relative abundance and during linear and stationary phases, *Algimonas* dominated the bacterial community.

**Figure 8:**
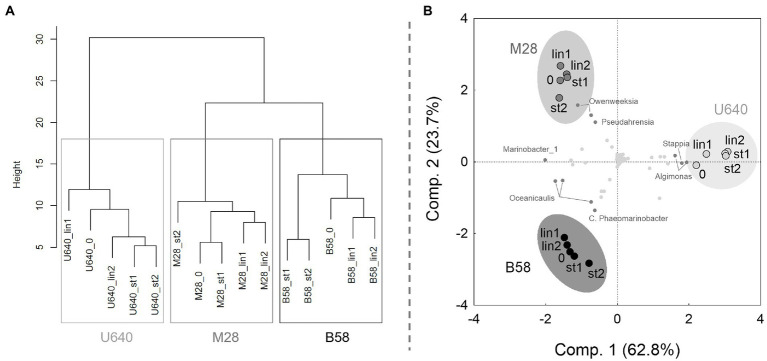
Statistical analysis of the microbiome data from the bubble column experiment. **(A)** Dendrogram and **(B)** PCA of the normalized bacterial community composition data. All sequenced OTUs were included in the calculations and are shown as grey dots in the PCA plot, and OTUs with largest impact are shown as dark gray dots with bacterial genus name. 0: start of the cultivation; lin, linear growth phase; st, stationary phase. Numbers 1 and 2 refer to the two biological replicates.

Hierarchical clustering of the microalgal culture samples collected during the cultivation phases, based on their bacterial community structures, resulted as expected in three main clusters, one for each microalgal strain (Figure 8A). The biological replicate samples collected at the linear and stationary phases did not consistently cluster together, however, suggesting that the bacterial composition remained stable throughout the experiment. These observations are supported by the PCA of the bacterial community compositions, where the samples from each strain form very tight and distinct cluster (Figure 8B).

## 4. Discussion

We investigated the bacterial community composition for nine microalgal strains in stock culture maintained in two different growth media, and for three *Phaeodactylum* strains during lab-scale batch cultivation in bubble columns, to assess if the bacterial composition is related to specific factors such as microalgal taxonomy, origin of isolation, growth medium, or growth phases.

### 4.1. Bacterial community composition of microalgal stock cultures

Bacterial genera of the classes *Alphaproteobacteria*, *Gammaproteobacteria*, and *Bacteroidetes* dominated the bacterial communities of all stock culture samples. These three bacterial classes are known to be the most prominent heterotrophic organisms in marine surface waters and are repeatedly found associated with microalgae in natural systems (Amin et al., 2012; Buchan et al., 2014). Statistical analysis of the bacterial community structures at the genus level showed that the bacterial community structures were more similar within each microalgal genus group (*Phaeodactylum*, *Entomoneis*, and *Tetraselmis*) than between genera, regardless of culture medium (Conway or NORCE). Similar results were found by Ling et al. (2020) who investigated the bacterial community composition of seven microalgae, including three strains of *Isochrysis galbana*, two strains of *Thalassiosira pseudonana*, and two strains of *Nannochloropsis oceanica*. The bacterial community composition at the class level was dominated by *Alphaproteobacteria* and *Gammaproteobacteria* in all seven strains, but at the genus level, stronger differences in the bacterial community composition were observed between the three microalgal species than between the different strains of the same species (Ling et al., 2020). However, our results showed that the bacterial community composition was affected by the growth medium as well, which was primarily apparent within the *Phaeodactylum* genus group. In line with the bacterial community composition, also hierarchical clustering of the microalgal and bacterial cell concentrations revealed distinct clusters for each of the three microalgal genera and distinct grouping by growth medium within the *Phaeodactylum* genus. The significant correlation between the two distance matrices (community composition and cell count) suggests that the two measures corroborate each other. Hence, the different microalgae and their associated bacterial populations seem to respond distinctly to the prevailing growth conditions.

Most of the strains of each microalgal genus group originate from different habitats or were isolated at different locations and times (Table 1). Hence, the bacterial composition does not seem to be related to the time of isolation or origin of the microalgae (Supplementary Figure S1, Supplementary section). All strains had been maintained in Conway medium and at the same cultivation conditions for several years (4–23 years), indicating that the bacterial community structure was not consistently shaped by the growth medium or cultivation conditions, but it appears to be somewhat linked to the microalgal genus. Bacteria were detected in the axenically obtained *Phaeodactylum* strain (U640) with the same quantities as in the other *Phaeodactylum* cultures and were most probably introduced during transfer of the culture. Hence, culture transfer could possibly have been the source for bacterial populations present in other cultures as well.

In Conway medium, the similarity in bacterial community composition for the different strains within each genus group was more distinct compared to NORCE medium. In NORCE medium, the bacterial communities shifted greatly and to very different community structures for the different *Phaeodactylum* strains. Only strains M26 and M29 displayed nearly the same community structure, but with very different bacterial genera compared to their Conway culture and to those of the other *Phaeodactylum* strains. For *Entomoneis* strains, the bacterial community shifted somewhat from Conway to NORCE, whereas the two *Tetraselmis* species had almost identical bacterial community composition as in Conway medium. *Phaeodactylum* and *Entomoneis* strains had been maintained in NORCE medium longer than the two *Tetraselmis* strains. Hence, the time differences of strains being maintained in NORCE medium might explain why the *Phaeodactylum* and *Entomoneis* strains had the greatest changes in bacterial community structure between Conway and NORCE and indicate that a shift in bacterial community composition probably occurs over a period of several months.

Although specific bacterial genera and their abundances were responsible for the clustering of the different microalgal genera, none of the bacterial genera were exclusively related to a specific microalgal genus or growth medium, but most of them were also present in the other cultures, in minor amounts. Thus, these findings imply that different microalgal species or genera could create distinct conditions that select for dominance of specific bacteria. The fact that the development of the bacterial community composition might at least to some extent be microalgal species or genus specific has been suggested before (Sapp et al., 2007; Ling et al., 2020) and could be related to the organic carbon and other metabolites that are released by the different microalgae. The main carbon source for bacteria in microalgal cultures is the DOC released by the microalgae and particulate and dissolved organic matter from senescent or lysed microalgal cells, which differ greatly between different microalgal species (Grossart et al., 2005). These compounds might constitute a selection pressure and might induce the development of adapted bacteria (Schäfer et al., 2002). However, Gouveia et al. (2019) investigated the bacterial community composition in 12 different strains of the green algae *Botryococcus braunii*, covering three different races, which secrete different hydrocarbons. In this study, the specific bacterial communities did not correlate to the different types of hydrocarbons produced by the different races, but each strain seemed to have its own specific bacterial community (Gouveia et al., 2019).

The development of different bacterial communities for some strains when cultured in NORCE medium over a longer time period indicates that the relationship of the bacterial community to the microalgal genus is not absolute, but that other factors such as the composition of growth medium can also affect the formation of the bacterial community structure. The two media were different in nitrogen and phosphate concentration, phosphate source, and micronutrient source and concentration, which all could alter microalgal and bacterial physiology and abundance. The bacterial genus *Marinobacter*_1 (*Gammaproteobacteria*) increased in relative abundance in four of the *Phaeodactylum* strains and in the two *Entomoneis* strains maintained in NORCE medium, although to different degrees. Hence, the medium composition could be beneficial for the development of this bacterial genus. The genus *Marinobacter* is ubiquitously found in close association to microalgal cultures and interestingly, is known to produce siderophores, which are known to increase the microalgal capacity for iron uptake (Bolch et al., 2011; Amin et al., 2012). In NORCE medium, the iron concentration was much higher compared to Conway medium; however, also the chelating agent in the medium is important in determining the bioavailability of the iron to the microalgae (Kean et al., 2015). In Conway, the chelating agent was EDTA and in NORCE medium DTPA, which could have an impact on the actual bioavailability of the iron. The fact that the nutrient concentration of the growth medium affects bacterial abundance and composition has been shown before. In a study of Grossart and Simon (2007), addition of a natural bacterial community to an axenic culture of *Thalassiosira rotula* affected the growth of the diatom differently in two growth media with different nutrient concentrations (Grossart and Simon, 2007) and Moejes et al. (2017) found that the bacterial community in *Phaeodactylum* cultures developed differently in two media with different nutrient content. In their study, they used *P. tricornutum* strain CCAP 1052/1B, which is a deposition of strain U640, and interestingly the bacterial community composition was very different to the ones observed in our study. In a study of Lian et al. (2022) the bacterial community composition in different pilot-scale photobioreactors producing *Nannochloropsis* sp. was shown to correlate significantly with the nitrate concentration, and Tait et al. (2019) observed a shift in the bacterial community composition associated with a freshwater *Chlorella sp*. isolate, when cultured in three growth media with different nitrogen and phosphorous concentrations. Furthermore, they could show that specific bacterial isolates could enhance growth of *C. vulgaris*, but only in one of the three media, supporting the theory that bacterial impact on microalgal growth is not only species-specific but also affected by environmental conditions (Tait et al., 2019).

### 4.2. Bacterial community composition during batch cultivation

During batch cultivation in bubble columns, the three *Phaeodactylum* strains grew to high biomass concentrations and at the same time also bacterial abundance increased from start of the experiment to stationary phase. Strain U640 grew slower and reached lower biomass concentrations compared to strains B58 and M28. Furthermore, its color was somewhat lighter, and the cells flocculated during the batch experiment. At the same time, a stronger increase in bacterial concentration occurred, compared to the other two strains which can indicate that strain U640 was somewhat stressed during the batch cultivation process.

During batch cultivation, each strain expressed a unique and stable bacterial composition which was very similar to that of the respective NORCE stock culture but differed in the relative abundances of the most dominant bacteria. The greatest change in bacterial community structure from stock culture through batch cultivation was observed in strain U640. The bacterial genus *Algimonas* was present in only low amounts in the stock culture, had increased slightly in the beginning of the experiment, and finally dominated the population during batch cultivation. During biomass upscaling and actual batch experiment, growth conditions in terms of irradiance and temperature but also pH and shear force deviated from those for the stock cultures, which could have had an impact on the bacterial populations and cause distinct bacterial genera to dominate. During inoculum production, cultures were maintained at higher temperatures and different light condition (15°C, 50 μmol m^−2^ s^−1^) compared to stock cultures (7°C, 35 μmol m^−2^ s^−1^), and during the batch experiment, temperature was increased to 24°C, illumination was continuous, and the cultures were aerated with 1% CO_2_ enriched air which influenced the pH in the culture and exerts shear stress on the organisms. It is possible that the prevalent conditions and a stressed algal culture (U640) were beneficial for *Algimonas* which therefore could outcompete the remaining bacteria in this culture. It is also possible that *Algimonas* had detrimental effects on strain U640 and thus caused the decreased performance of this strain. Negative effect of specific bacterial genera on microalgal growth was shown by Tait et al. (2019) where addition of *Pseudomonas* sp. to an axenic *C. vulgaris* culture resulted in a decrease in OD by 86%. Furthermore, it has been shown that specific bacteria and their secreted components can induce microalgal flocculation (Wang et al., 2012; Lee et al., 2013; Lian et al., 2018). In the other two *Phaeodactylum* cultures, *Algimonas* could not be detected at the start of the experiment but was present with 0.06–1.81% at the end of the cultivation. The fact that *Algimonas* did not increase to higher numbers, although present in the other cultures, could be because the starting numbers were too low or that the stable community of other bacteria prevented them from proliferating. Applying the different bacterial communities to axenic cultures of the respective other *Phaeodactylum* strains could give further insight on the actual impact and the possible cause vs. consequence of the different bacterial communities on the different strains. Specific positive and negative effect of different bacterial species on microalgal growth has been demonstrated before (Tait et al., 2019; Ling et al., 2020; Lian et al., 2021).

During the batch experiment in 80 ml bubble columns, the bacterial community structure remained rather stable throughout the 9–10 days of cultivation for each *Phaeodactylum* strain. Other studies investigating bacterial communities in microalgal cultures across time revealed very different results and different bacterial dynamics. Some found the bacterial composition to be stable during cultivation (Lupette et al., 2016), while others observed a strong shift in bacterial structure (Moejes et al., 2017), and some studies found stable bacterial populations for some microalgal species and changes in bacterial composition for other species (Schäfer et al., 2002). Usually, when growing microalgae in NORCE medium, stationary phase is reached due to light and not nutrient limitation, as the high nutrient concentrations allow the microalgae to grow to very high densities (>3 g L^−1^, authors observation). At these densities, light penetration into the culture becomes limiting, leading to a decrease in growth before nutrients are used up. In many other lab-scale experiments, the chosen medium had much lower nutrient concentrations, comparable to those of Conway medium. Hence, microalgae often reached stationary phase due to nutrient limitation during batch cultivation. If nutrients become limiting, microalgal cells become stressed, senescent, and finally die. This causes more and different organic carbon fractions to become available for the bacteria, which again impacts the bacterial abundance and community structure. This has also been observed in field studies, where bacterial growth is often highest at the end of phytoplankton blooms (Grossart et al., 2005). Also, the chemical nature and concentration of the organic carbon compounds released by the microalgae vary with the physiological status of the algae, affecting their stoichiometry (such as C:N:P ratio) and bioreactivity which, in turn, affects the metabolic activity and proliferation of the bacteria (Buchan et al., 2014).

### 4.3. Algae-bacteria interaction in culture

In natural aquatic environments, bacteria generally outnumber microalgal cells by 100–1,000 times (Wang et al., 2016), with around 10^6^ and 10^2^–10^5^ cells mL^−1^ for bacteria and microalgae, respectively (Cole, 1982). In cultivation systems, the additionally provided nutrients and irradiance increase microalgal growth rates, which often decreases the bacteria to microalgae ratio to 10 (Wang et al., 2016). In the different stock cultures investigated in this study, the bacteria-to-algae ratios varied greatly between 3 and 141, with strong differences between the different genera and for some also between the media. These rather high bacteria-to algae-ratios are probably due to the non-ideal cultivation conditions for the stock culture (low light, low temperature) which were chosen to minimize the need for culture transfer. For most strains, the bacterial concentration was also lower in NORCE medium than in Conway medium which could indicate that the strains in Conway medium were more stressed due to lower nutrient content which can promote bacterial growth due to the release of DOC. During batch cultivation, the bacteria-to-algae ratio decreased with increasing cultivation time from 28 and 24 at the start of cultivation to 8 and 7 at the end of cultivation for strains M28 and B58, respectively. For strain U640 however, the bacteria-to-algae ratio increased from 15 to 41, which supports the theory that this strain was stressed during batch cultivation.

In nature, bacteria and microalgae form an interactive relationship where bacteria receive DOC from microalgae and the bacteria recycle nutrients which get accessible for the microalgae, or provide CO_2_, vitamins or other compounds which enable microalgae to grow. In laboratory cultures, however, the relationship between bacteria and algae remains more enigmatic. In general, the growth medium provides the microalgae with all essential nutrients, and CO_2_ and irradiance can be provided accordingly. Nevertheless, several studies have shown that bacteria also affect microalgal growth in culture, and often the addition of bacteria leads to increased growth of the microalgae (Park et al., 2008; Biondi et al., 2017; Berthold et al., 2019; Ling et al., 2020; Chorazyczewski et al., 2021). However, this effect has also been shown to be species-specific and related to the physiological stage of the algae (Grossart et al., 2005). Further investigations on how specific bacterial communities develop over time in different microalgal cultures combined with varying growth conditions could provide a better understanding of algae-bacteria relationships in culture and how much influence microalgae have in modulating the bacterial community.

## 5. Conclusion

The bacterial community composition was distinct for three different microalgal genera (*Phaeodactylum*, *Entomoneis*, and *Tetraselmis*) but similar for different strains of the same genus for nine microalgal stock cultures, after having been maintained at the same growth conditions for several years (Conway medium, low light, and low temperature). The isolation origin of the different strains did not seem to be related to the prevalent bacterial population in the cultures. Several months after being transferred to a different growth medium with high nutrient concentration (NORCE), the bacterial community structure had changed for five *Phaeodactylum* strains, with separate bacterial community structures for the different strains. During batch cultivation of three *Phaeodactylum* strains, the bacterial communities remained similar to their respective stock culture microbiome but with some changes in relative abundance of some bacterial genera. The overall community structure remained balanced during batch cultivation with cultures being dominated by the same 1–2 different bacterial genera. These findings support results of other studies showing that bacterial community compositions can be distinct for different microalgal genera, but also dependent on other cultivation parameters. The dominance of certain bacterial genera associated with specific microalgal strains suggests that the microalgae to some degree have an influence on the development of the bacterial communities in different settings.

## Data availability statement

The sequencing data presented in this study can be found in online repositories. The names of the repository/repositories and accession number(s) can be found at: https://www.ebi.ac.uk/ena/browser/view/PRJEB46865, PRJEB46865.

## Author contributions

PS, OM, and DK designed the research. PS and SJ performed the research. PS, SJ, OM, and PP analyzed the data. PS wrote the paper with contribution from OM and PP. SJ, OM, PP, and DK revised the paper. All authors contributed to the article and approved the submitted version.

## Funding

This work was supported by the Algae2future project (A2F) and has received funding from the Research Council of Norway (grant number 267872).

## Conflict of interest

The authors declare that the research was conducted in the absence of any commercial or financial relationships that could be construed as a potential conflict of interest.

## Publisher’s note

All claims expressed in this article are solely those of the authors and do not necessarily represent those of their affiliated organizations, or those of the publisher, the editors and the reviewers. Any product that may be evaluated in this article, or claim that may be made by its manufacturer, is not guaranteed or endorsed by the publisher.

## References

[ref1] Aiyar P. Schaeme D. García-Altares M. Carrasco Flores D. Dathe H. Hertweck C.. (2017). Antagonistic bacteria disrupt calcium homeostasis and immobilize algal cells. Nat. Commun. 8:1756. doi: 10.1038/s41467-017-01547-8, PMID: 29170415 PMC5701020

[ref2] Amin S. A. Parker M. S. Armbrust E. V. (2012). Interactions between diatoms and bacteria. Microbiol. Mol. Biol. Rev. 76, 667–684. doi: 10.1128/mmbr.00007-12, PMID: 22933565 PMC3429620

[ref3] Beckers B. Thijs S. Truyens S. Weyens N. Boerjan W. Vangronsveld J.. (2016). Performance of 16s rDNA primer pairs in the study of Rhizosphere and Endosphere bacterial microbiomes in Metabarcoding studies study site description and sampling. Front. Microbiol. 7:650. doi: 10.3389/fmicb.2016.00650, PMID: 27242686 PMC4865482

[ref4] Berthold D. E. Shetty K. G. Jayachandran K. Laughinghouse H. D. Gantar M. (2019). Enhancing algal biomass and lipid production through bacterial co-culture. Biomass Bioenergy 122, 280–289. doi: 10.1016/j.biombioe.2019.01.033

[ref5] Biondi N. Cheloni G. Rodolfi L. Viti C. Giovannetti L. Tredici M. R. (2018). *Tetraselmis suecica* F&M-M33 growth is influenced by its associated bacteria. Microb. Biotechnol. 11, 211–223. doi: 10.1111/1751-7915.12865, PMID: 29105335 PMC5743789

[ref6] Biondi N. Cheloni G. Tatti E. Decorosi F. Rodolfi L. Giovannetti L.. (2017). The bacterial community associated with *Tetraselmis suecica* outdoor mass cultures. J. Appl. Phycol. 29, 67–78. doi: 10.1007/s10811-016-0966-5

[ref7] Bolch C. J. S. Subramanian T. A. Green D. H. (2011). The toxic dinoflagellate *Gymnodinium catenatum* (Dinophyceae) requires marine bacteria for growth. J. Phycol. 47, 1009–1022. doi: 10.1111/j.1529-8817.2011.01043.x, PMID: 27020182

[ref8] Buchan A. LeCleir G. R. Gulvik C. A. González J. M. (2014). Master recyclers: features and functions of bacteria associated with phytoplankton blooms. Nat. Rev. Microbiol. 12, 686–698. doi: 10.1038/nrmicro3326, PMID: 25134618

[ref9] Callahan B. J. McMurdie P. J. Rosen M. J. Han A. W. Johnson A. J. A. Holmes S. P. (2016). DADA2: high-resolution sample inference from Illumina amplicon data. Nat. Methods 13, 581–583. doi: 10.1038/nmeth.3869, PMID: 27214047 PMC4927377

[ref10] Chorazyczewski A. M. Huang I. S. Abdulla H. Mayali X. Zimba P. V. (2021). The influence of bacteria on the growth, lipid production, and extracellular metabolite accumulation by *Phaeodactylum tricornutum* (*Bacillariophyceae*). J. Phycol. 57, 931–940. doi: 10.1111/jpy.13132, PMID: 33454979

[ref11] Cole J. J. (1982). Interactions between bacteria and algae in aquatic ecosystems. Annu. Rev. Ecol. Syst. 13, 291–314. doi: 10.1146/annurev.es.13.110182.001451

[ref12] Fernández F. G. A. Reis A. Wijffels R. H. Barbosa M. Verdelho V. Llamas B. (2021). The role of microalgae in the bioeconomy. New Biotechnol. 61, 99–107. doi: 10.1016/j.nbt.2020.11.011, PMID: 33249179

[ref13] Gloor G. B. Reid G. (2016). Compositional analysis: a valid approach to analyze microbiome high-throughput sequencing data. Can. J. Microbiol. 62, 692–703. doi: 10.1139/cjm-2015-0821, PMID: 27314511

[ref14] González-González L. M. De-Bashan L. E. (2021). Toward the enhancement of microalgal metabolite production through microalgae–bacteria consortia†. Biology 10, 1–20. doi: 10.3390/biology10040282, PMID: 33915681 PMC8065533

[ref15] Gouveia J. D. Lian J. Steinert G. Smidt H. Sipkema D. Wijffels R. H.. (2019). Associated bacteria of *Botryococcus braunii* (*Chlorophyta*). PeerJ 7:e6610. doi: 10.7717/peerj.6610, PMID: 30944776 PMC6441321

[ref16] Grossart H. Levold F. Allgaier M. Simon M. Brinkhoff T. (2005). Marine diatom species harbour distinct bacterial communities. Environ. Microbiol. 7, 860–873. doi: 10.1111/j.1462-2920.2005.00759.x, PMID: 15892705

[ref17] Grossart H. Simon M. (2007). Interactions of planktonic algae and bacteria: effects on algal growth and organic matter dynamics. Aquat. Microb. Ecol. 47, 163–176. doi: 10.3354/ame047163

[ref18] Kean M. A. Delgado E. B. Mensink B. P. Bugter M. H. J. (2015). Iron chelating agents and their effects on the growth of *Pseudokirchneriella subcapitata*, *Chlorella vulgaris*, *Phaeodactylum tricornutum* and *Spirulina platensis* in comparison to Fe-EDTA. J. Algal Biomass Utln. 6, 56–73. doi: 10.1000/xyz999

[ref19] Knuckey R. M. Brown M. R. Barrett S. M. Hallegraeff G. M. (2002). Isolation of new nanoplanktonic diatom strains and their evaluation as diets for juvenile Pacific oysters (Crassostrea gigas). Aquaculture 211, 253–274. doi: 10.1016/S0044-8486(02)00010-8

[ref20] Le Chevanton M. Garnier M. Bougaran G. Schreiber N. Lukomska E. Bérard J. B.. (2013). Screening and selection of growth-promoting bacteria for *Dunaliella* cultures. Algal Res. 2, 212–222. doi: 10.1016/j.algal.2013.05.003

[ref21] Lee J. Cho D. H. Ramanan R. Kim B. H. Oh H. M. Kim H. S. (2013). Microalgae-associated bacteria play a key role in the flocculation of *Chlorella vulgaris*. Bioresour. Technol. 131, 195–201. doi: 10.1016/j.biortech.2012.11.130, PMID: 23347927

[ref22] Lian J. Schimmel P. Sanchez-Garcia S. Wijffels R. H. Smidt H. Sipkema D. (2021). Different co-occurring bacteria enhance or decrease the growth of the microalga *Nannochloropsis* sp. CCAP211/78. Microb. Biotechnol. 14, 1159–1170. doi: 10.1111/1751-7915.13784, PMID: 33683803 PMC8085966

[ref23] Lian J. Steinert G. de Vree J. Meijer S. Heryanto C. Bosma R.. (2022). Bacterial diversity in different outdoor pilot plant photobioreactor types during production of the microalga *Nannochloropsis* sp. CCAP211/78. Appl. Microbiol. Biotechnol. 106, 2235–2248. doi: 10.1007/s00253-022-11815-3, PMID: 35166894 PMC8930801

[ref24] Lian J. Wijffels R. H. Smidt H. Sipkema D. (2018). The effect of the algal microbiome on industrial production of microalgae. Microb. Biotechnol. 11, 806–818. doi: 10.1111/1751-7915.13296, PMID: 29978601 PMC6116740

[ref25] Ling T. Zhang Y. F. Cao J. Y. Xu J. L. Kong Z. Y. Zhang L.. (2020). Analysis of bacterial community diversity within seven bait-microalgae. Algal Res. 51:102033. doi: 10.1016/j.algal.2020.102033

[ref26] Lupette J. Lami R. Krasovec M. Grimsley N. Moreau H. Piganeau G.. (2016). Marinobacter dominates the bacterial community of the *Ostreococcus tauri* phycosphere in culture. Front. Microbiol. 7, 1–14. doi: 10.3389/fmicb.2016.01414, PMID: 27656176 PMC5013054

[ref001] Marotz C. Sharma A. Humphrey G. Gottel N. Daum C. Gilbert J. A.. (2019). Triplicate PCR reactions for 16S rRNA gene amplicon sequencing are unnecessary. Biotechniques 67, 29–32. doi: 10.2144/btn-2018-0192 31124709 PMC7030937

[ref27] Moejes F. Succurro A. Popa O. Maguire J. Ebenhöh O. (2017). Dynamics of the bacterial community associated with *Phaeodactylum tricornutum* cultures. PRO 5:77. doi: 10.3390/pr5040077

[ref28] Park Y. Je K. W. Lee K. Jung S. E. Choi T. J. (2008). Growth promotion of Chlorella ellipsoidea by co-inoculation with *Brevundimonas* sp. isolated from the microalga. Hydrobiologia 598, 219–228. doi: 10.1007/s10750-007-9152-8

[ref29] Paul C. Pohnert G. (2011). Interactions of the algicidal bacterium Kordia algicida with diatoms: regulated protease excretion for specific algal lysis. PLoS One 6:e21032. doi: 10.1371/journal.pone.0021032, PMID: 21695044 PMC3117869

[ref30] Prestegard S. K. Oftedal L. Coyne R. T. Nygaard G. Skjærven K. H. Knutsen G.. (2009). Marine benthic diatoms contain compounds able to induce leukemia cell death and modulate blood platelet activity. Mar. Drugs 7, 605–623. doi: 10.3390/md7040605, PMID: 20098602 PMC2810217

[ref31] R Core Team (2020). R: A Language and Environment for Statistical Computing. R Foundation for Statistical Computing, Vienna, Austria.

[ref32] Sapp M. Schwaderer A. S. Wiltshire K. H. Hoppe H. G. Gerdts G. Wichels A. (2007). Species-specific bacterial communities in the phycosphere of microalgae? Microb. Ecol. 53, 683–699. doi: 10.1007/s00248-006-9162-5, PMID: 17264999

[ref33] Schäfer H. Abbas B. Witte H. Muyzer G. (2002). Genetic diversity of “satellite” bacteria present in cultures of marine diatoms. FEMS Microbiol. Ecol. 42, 25–35. doi: 10.1111/j.1574-6941.2002.tb00992.x, PMID: 19709263

[ref34] Seymour J. R. Amin S. A. Raina J. B. Stocker R. (2017). Zooming in on the phycosphere: the ecological interface for phytoplankton-bacteria relationships. Nat. Microbiol. 2:17065. doi: 10.1038/nmicrobiol.2017.65 28555622

[ref35] Steinrücken P. Erga S. R. Mjøs S. A. Kleivdal H. Prestegard S. K. (2017). Bioprospecting North Atlantic microalgae with fast growth and high polyunsaturated fatty acid (PUFA) content for microalgae-based technologies. Algal Res. 26, 392–401. doi: 10.1016/j.algal.2017.07.030, PMID: 28989862 PMC5614095

[ref36] Tait K. White D. A. Kimmance S. A. Tarran G. Rooks P. Jones M.. (2019). Characterisation of bacteria from the cultures of a chlorella strain isolated from textile wastewater and their growth enhancing effects on the axenic cultures of *Chlorella vulgaris* in low nutrient media. Algal Res. 44:101666. doi: 10.1016/j.algal.2019.101666

[ref37] Walne P. R. (1970). Studies on the food value of nineteen genera of algae to juvenile bivalves of the genera *Ostrea*, *Crassostrea*, *Mercenaria* and *Mytilus*. Fish. Invest. Lond. Ser. 2, 1–62.

[ref38] Wang H. Hill R. T. Zheng T. Hu X. Wang B. (2016). Effects of bacterial communities on biofuel-producing microalgae: stimulation, inhibition and harvesting. Crit. Rev. Biotechnol. 36, 341–352. doi: 10.3109/07388551.2014.961402, PMID: 25264573

[ref39] Wang H. Laughinghouse H. D. Anderson M. A. Chen F. Willliams E. Place A. R.. (2012). Novel bacterial isolate from permian groundwater, capable of aggregating potential biofuel-producing microalga *Nannochloropsis oceanica* IMET1. Appl. Environ. Microbiol. 78, 1445–1453. doi: 10.1128/AEM.06474-11, PMID: 22194289 PMC3294481

[ref002] Wen C. Wu L. Qin Y. Van Nostrand J. D. Ning D. Sun B.. (2017). Evaluation of the reproducibility of amplicon sequencing with Illumina MiSeq platform. PLoS One 12, 1–20. doi: 10.1371/journal.pone.0176716 PMC5409056 28453559

