# Supplementary material for: A closer look into the microbiome of microalgal cultures
Source: Front Microbiol. 2023 Jan 26;14:1108018. doi: 10.3389/fmicb.2023.1108018 (PMC9908576; doi:10.3389/fmicb.2023.1108018)
Supplement: Supplementary file 1 [file Image_1.pdf]

Supplementary Material

A closer look into the microbiome of microalgal cultures

Pia Steinrücken*, Steve Jackson, Oliver Müller, Pål Puntervoll, Dorinde M.M. Kleinegris

*** Correspondence:** Pia Steinrücken: pias@norceresearch.no

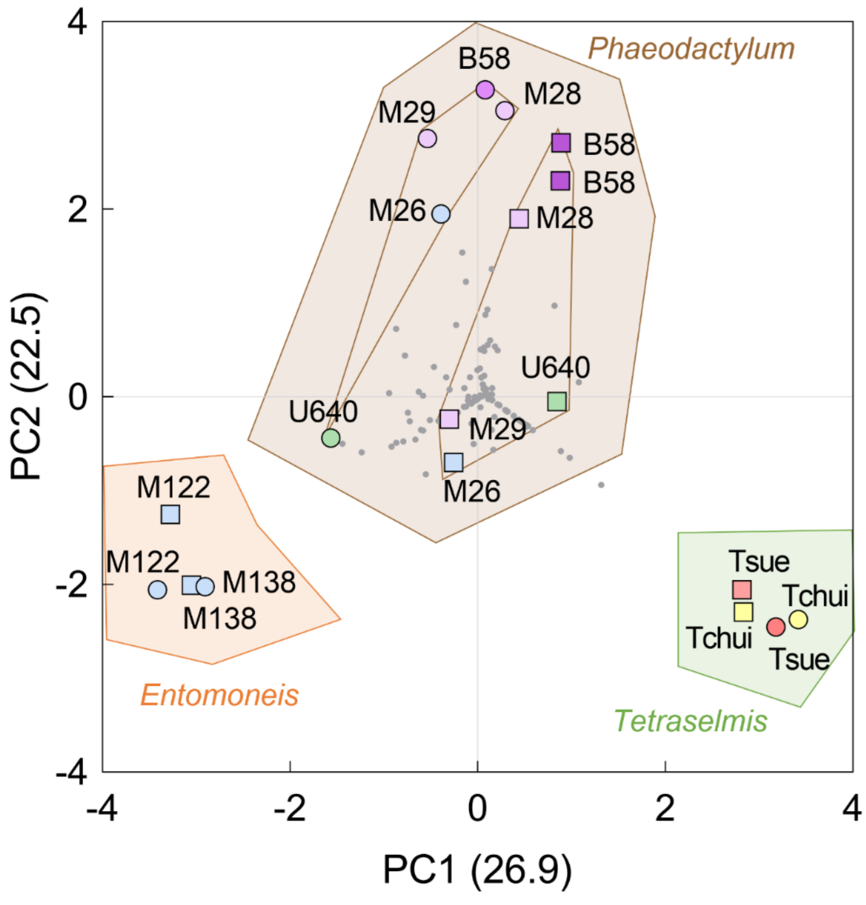

Supplementary Figure S1. Principal component analysis (PCA) of the normalized bacterial community composition data for 18 microalgal stock cultures (nine strains, two growth media). All sequenced OTUs were included in the calculations and are shown as grey dots. Circles indicate Conway and squares NORCE medium. For *Phaeodactylum* stock cultures inner connecting lines indicate two distinct subclusters defined by culture medium (Conway or NORCE). Colors for circles and squares indicate different sampling habitats with blue: Store Lungegårdsvann, Bergen; green: Arctic; light purple: Puddefjorden, Bergen, 2014; dark purple: Puddefjorden, Bergen, 1997; grey: England; yellow: Scotland; red: Italy.

frontiers
