# Supplementary material for: A closer look into the microbiome of microalgal cultures
Source: Front Microbiol. 2023 Jan 26;14:1108018. doi: 10.3389/fmicb.2023.1108018 (PMC9908576; doi:10.3389/fmicb.2023.1108018)
Supplement: Supplementary file 2 [file Table_1.pdf]

Supplementary Material

A closer look into the microbiome of microalgal cultures

Pia Steinrücken[*], Steve Jackson, Oliver Müller, Pål Puntervoll, Dorinde M.M. Kleinegris

*** Correspondence:** Pia Steinrücken: pias@norceresearch.no

Supplementary Table S1. Final nutrient concentration in Conway and NORCE medium. In the NORCE medium micronutrients were added through a fertilizer (YaraTera REXOLIN APN, Bor 0.85%, Copper 0.25%, Iron 6%, Manganese 2.4%, Molybdenum 0.25%, Zink 1.3%), Cu, Mn and Zn are chelated with EDTA, and Fe with DTPA. In Conway micronutrients are chelated with EDTA.

**Conway**	**Molarity (mmol/L)**	**NORCE**	**Molarity (mmol/L)**
**Macronutrients**		**Macronutrients**	
**Nitrate ($NaNO_3$)**	1.177	Nitrate ($NaNO_3$)	12.47
**Phosphate ($NaH_2PO_4 \cdot H_2O$)**	0.113	Phosphate (KH_2PO_4)	0.88
**Silicate ($Na_2SiO_3 \cdot 5H_2O$)**	0.141	Silicate ($Na_2SiO_3 \cdot 5H_2O$)	0.14
**Micronutrients**		**Trace mineral mix**	
**H_3BO_3**	0.543	Boron (B)	0.031
**$CoCl_2 \cdot 6H_2O$**	0.00008		
**$CuSO_4 \cdot 5H_2O$**	0.00008	Copper (Cu)	0.002
**$FeCl_3 \cdot 6H_2O$**	0.008	Iron (Fe)	0.043
**$MnCl_2 \cdot 4H_2O$**	0.002	Manganese (Mn)	0.017
**$(NH_4)_6Mo_7O_{24} \cdot 4H_2O$**	0.0000073	Molybdenum (Mo)	0.001
**$ZnCl_2$**	0.00015	Zink (Zn)	0.008
**Vitamins**		**Vitamins**	
**B1**	0.000297	B1	0.000297
**B12**	0.0000037	B12	0.0000037
**Biotin**	0.000021	Biotin	0.000021
