# Supplementary material for: A closer look into the microbiome of microalgal cultures
Source: Front Microbiol. 2023 Jan 26;14:1108018. doi: 10.3389/fmicb.2023.1108018 (PMC9908576; doi:10.3389/fmicb.2023.1108018)
Supplement: Supplementary file 3 [file Table_2.pdf]

frontiers

Supplementary Material

A closer look into the microbiome of microalgal cultures

Pia Steinrücken*, Steve Jackson, Oliver Müller, Pål Puntervoll, Dorinde M.M. Kleinegris

*** Correspondence:** Pia Steinrücken: pias@norceresearch.no

Supplementary Table S2. Irradiance (µmol m^{-2} s^{-1}) and culture densities (OD 750, average of two technical replicates) during batch cultivation in bubble columns of three *Phaeodactylum* strains (duplicates). Strains were grown in a Multi-Cultivator MC 1000-OD until stationary phase was reached, and irradiance was increased progressively with increasing OD 750.

Strain		Day 0	Day 1	Day 2	Day 3	Day 4	Day 5	Day 6	Day 7	Day 8	Day 9	Day 10
**B58_1**	Irradiance	30	30	30	60	60	200	300	500	500	500	500
	OD 750	~ 0.1	0.12	0.23	0.74	1.39	3.17	5.21	8.12	11.10	10.93	12.68
**B58_2**	Irradiance	30	30	30	60	60	200	300	500	500	500	500
	OD 750	~ 0.1	0.11	0.22	0.68	1.23	2.92	4.98	8.00	9.68	10.55	9.95
**M28_1**	Irradiance	30	30	30	60	60	200	300	500	500	500	500
	OD 750	~ 0.1	0.15	0.27	0.80	1.54	4.09	5.40	8.66	11.23	12.10	12.00
**M28_2**	Irradiance	30	30	30	60	60	200	300	500	500	500	500
	OD 750	~ 0.1	0.14	0.28	0.87	1.72	4.19	5.77	8.66	11.58	12.73	13.03
**U640_1**	Irradiance	30	30	30	60	60	100	200	300	500	500	-
	OD 750	~ 0.1	0.11	0.19	0.80	0.94	2.25	3.44	5.16	5.60	4.48	-
**U640_2**	Irradiance	30	30	30	60	60	100	200	300	500	500	-
	OD 750	~ 0.1	0.10	0.17	0.54	0.76	1.65	2.62	3.80	5.60	4.58	-
